# A novel olecranon-type tracheotomy and expansion forceps for single-person emergency airway access

**DOI:** 10.3389/fbioe.2025.1712809

**Published:** 2025-11-19

**Authors:** Liang Zhu, Fanhao Meng, Nenghao Jin, Lejun Xing, Yi Wang, Haizhong Zhang

**Affiliations:** 1 Medical School of Chinese PLA, Beijing, China; 2 Department of Stomatology, The First Medical Centre, Chinese PLA General Hospital, Beijing, China; 3 Department of Stomatology, Beijing Friend ship Hospital, Capital Medical University, Beijing, China; 4 Department of Stomatology, Beijing Tsinghua Changgung Hospital, School of Clinical Medicine, Tsinghua University, Beijing, China

**Keywords:** tracheotomy, airway management, emergency treatment, surgical instruments, cadaver

## Abstract

**Background:**

We developed olecranon-type tracheotomy and expansion forceps (OTEF) for emergency tracheotomy in patients experiencing acute airway obstruction. By using OTEF, medical rescue personnel can perform emergency tracheotomy more quickly and accurately on their own, while conventional tracheotomy requires the cooperation of two surgeons.

**Methods:**

In this study, 24 adult cadavers that had died within 24 h were randomly assigned to the OTEF or PDT groups. Tracheotomies were performed by the same physician, using the OTEF technique for the OTEF group and the percutaneous dilational tracheotomy technique for the PDT group. The collected data included basic cadaver characteristics, operative time, incision length, and intraoperative tracheal wall injuries.

**Results:**

In the OTEF group, the mean tracheotomy completion time was 96.83 ± 8.82 s, with a mean incision length of 13.67 ± 3.67 mm. In the PDT group, the mean tracheotomy completion time was 566.50 ± 47.14 s, and the mean incision length was 20.67 ± 4.76 mm. Compared with the PDT group, the OTEF group demonstrated significantly shorter operative times (P < 0.05) and smaller incision lengths (P < 0.05).

**Conclusion:**

OTEF enables efficient and minimally traumatic tracheostomies in emergency settings with minimal environmental and positioning requirements. In disaster sites or even on battlefields where medical personnel are in short supply, this device can enhance the battlefield rescue skills and emergency response capabilities of non-medical rescue workers, effectively alleviate rescue pressure and save the lives of the injured.

## 1 Background

The airway is a vital component of human physiology, and its obstruction represents a critical medical emergency. When conventional airway management techniques are unsuccessful or impractical, tracheotomy serves as a life-saving procedure, offering a direct route for ventilation. Traditionally, this invasive intervention has been performed by medical teams in controlled environments, such as hospitals or operating rooms. However, in specific critical situations—such as prehospital settings, remote areas, or emergencies with limited medical personnel—the availability of a full team cannot be guaranteed.

Current devices and instruments for emergency airway access, including traditional tracheotomy kits and cricothyrotomy tools, are predominantly designed for collaborative use by multiple individuals. Clinicians commonly employ two main types of tracheostomies: surgical tracheotomy and percutaneous dilational tracheotomy (Karlsson et al., 2022). Relevant studies indicate no significant differences in mortality or the incidence of major complications—such as respiratory distress, hemorrhagic shock, or tracheal stenosis—between these two procedures. However, percutaneous tracheotomy is associated with shorter operation times and reduced risks of wound infection (Putensen et al., 2014). Consequently, many clinical guidelines recommend percutaneous dilation tracheotomy when there are no contraindications (Karlsson et al., 2022; Putensen et al., 2014; Ciaglia et al., 1985). To further reduce operation times and complications, various improved surgical techniques have been developed, including guidewire dilating forceps (Griggs et al., 1990), trans laryngeal tracheotomy (Fantoni and Ripamonti, 1997), Ciaglia Blue Rhin (Byhahn et al., 2000), cricoid membrane puncture-directed tracheotomy (Chen et al., 2018; Chen et al., 2015), and quick-response tracheotomy (Graeme, 2016). These techniques often require a team of operators: one to stabilize the patient’s anatomy, another to make the incision, and a third to secure and confirm airway placement. Such multi-operator requirements make these methods unsuitable for single-operator scenarios, where an individual must independently manage every aspect of the procedure. There is evidence to support the argument that advanced airway management can be performed in the prehospital setting without delaying transfer to a trauma center. When performed by skilled emergency medical services providers, advanced airway management is associated with a significant decrease in mortality (Kovacs and Sowers, 2018). This limitation has driven interest in designing instruments specifically tailored for single-person use, enabling safe and efficient emergency tracheotomies even under adverse conditions.

To address these challenges, we developed a novel instrument specifically designed for single-person emergency tracheotomies, focusing on simplicity, precision, and ease of use. The device integrates features that streamline the procedure, minimize error margins, and enhance safety in high-pressure situations. For instance, it allows a single operator to stabilize the patient’s airway, make an incision, and insert a tracheotomy tube in a sequential and controlled manner. This design process was informed by the challenges commonly encountered during emergency airway management and aimed to overcome the limitations of existing methods.

## 2 Methods

### 2.1 Materials

**FIGURE 1:**
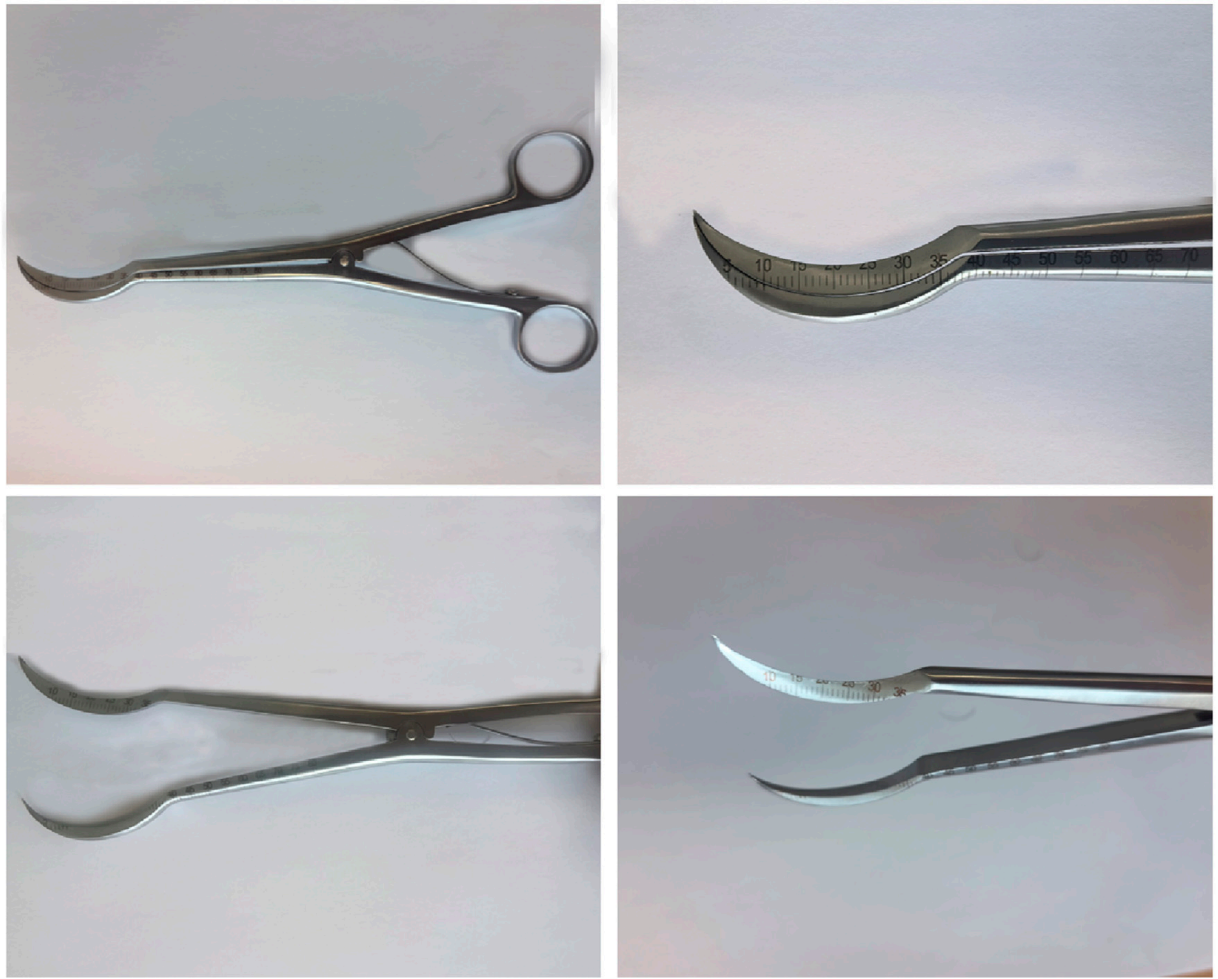
Olecranon-type tracheotomy and expansion forceps. One side features a sharp cutting edge, with scales marked from the tip to the side of the forceps body.

The research team independently designed an olecranon-type tracheotomy and expansion forceps, which has been submitted for invention patents (Patent number: ZL202011064693.2). The team aimed to integrate the tracheotomy and distraction instruments into a single device capable of performing both procedures. The team aimed to integrate a tracheotomy and distraction instrument into a single device capable of performing both tracheotomy and distraction procedures (Figure 1). The original design featured a straight tip. However, animal experiments revealed that the straight tip could easily injure the posterior tracheal wall during puncture. Moreover, the sharp tip posed a risk of scratching the tracheal mucosa during distraction. Consequently, the design was refined, resulting in the olecranon-shaped tracheotomy and distraction forceps. The device is fabricated from stainless steel, measures approximately 20 cm in length, and consists of a tip and a forceps handle. The front end of the forceps is sharp and arc-shaped. The inner surface of the arc features a sharp cutting edge, whereas the outer surface remains blunt. Graduated scales are engraved along the lateral aspect of the forceps, starting from the tip. When the forceps handle is closed, the jaws open, allowing the inner cutting edge to incise the trachea, thereby facilitating surgical manipulation. The device, fabricated from stainless steel, is approximately 20 cm in length and consists of a tip and a forceps handle. The front end of the forceps is sharp and arc-shaped. The inner surface of the arc features a sharp cutting edge, while the outer surface is blunt. Scales are engraved from the tip along the side of the forceps body. When the forceps handle is closed, the jaws open, allowing the inner cutting edge to incise the trachea, facilitating easier surgical manipulation.

### 2.2 Cadaver experiments

In this study, 24 non–formalin-fixed adult cadavers that had died within 24 h were enrolled. The cadavers were provided by the Body Donation Registration and Acceptance Station of the Chinese Academy of Medical Sciences and Peking Union Medical College. Each cadaver was intubated nasotracheally. A second physician conducted tracheal examinations with a fiberoptic bronchoscope to document intraoperative injuries during subsequent procedures.

**FIGURE 2:**
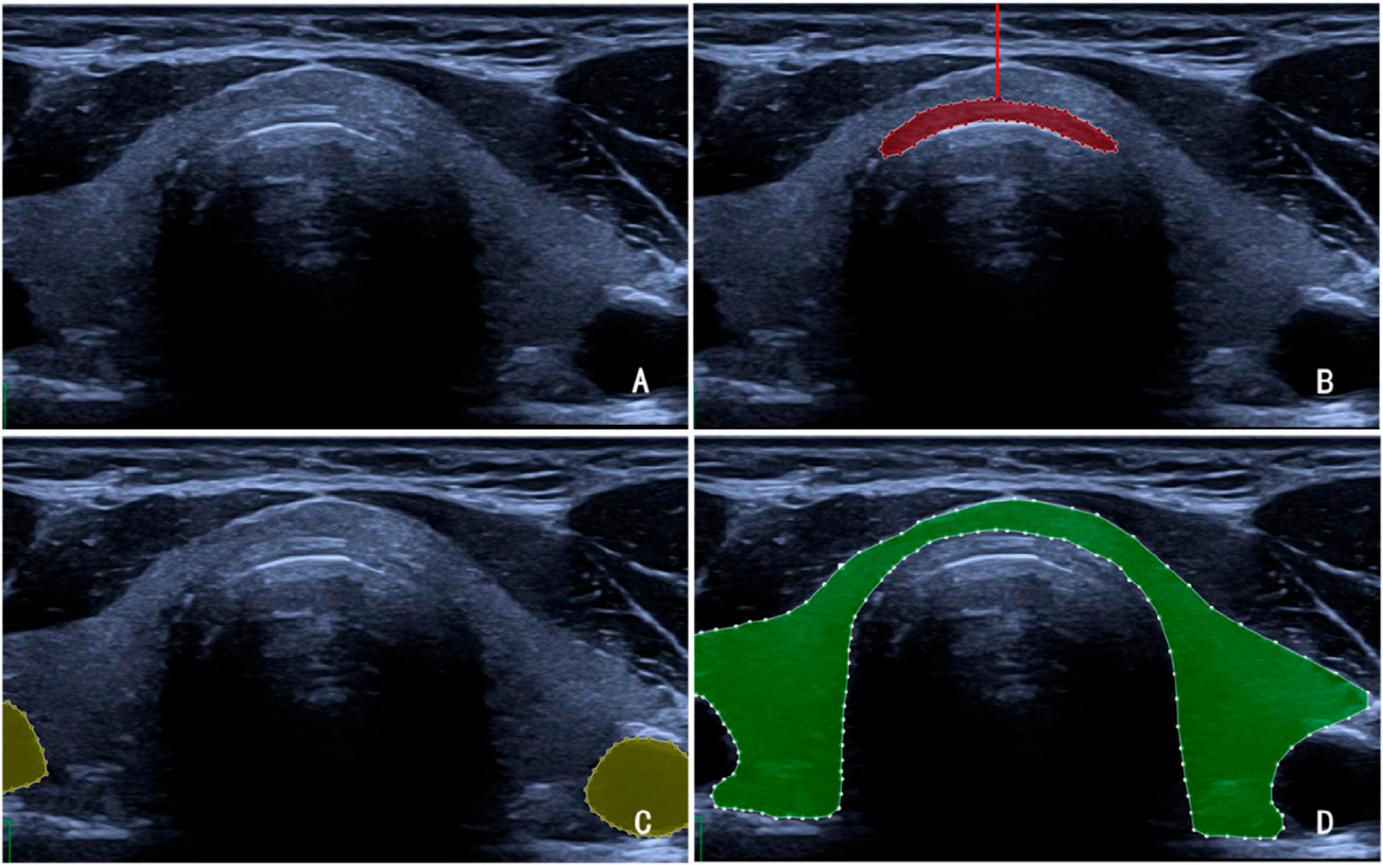
**(A)** Ultrasound image of a cadaver’s neck. **(B)** The red-shaded region in the figure denotes the tracheal rings, while the red line indicates the tracheal depth at that site, defined as the vertical distance from the trachea to the cervical skin surface. **(C)** The yellow-shaded region represents the vascular structures. **(D)** The green-shaded region represents the thyroid gland.

A coupling agent was applied to the cadavers’ necks, after which a portable ultrasonic diagnostic device was used to visualize the neck structures (Figure 2). This procedure allowed measurement of the distance from the anterior wall of the trachea to the skin surface and identification of the optimal tracheotomy site. The duration of the ultrasound assessment was recorded for each cadaver.

In the OTEF group, tracheotomy was performed using olecranon-type tracheotomy with expansion forceps, whereas in the PDT group, percutaneous dilational tracheotomy was performed. All procedures were conducted by the same surgeon to minimize operator variability. This experimental design enabled comparison of procedural feasibility, accuracy, and efficiency between the two tracheotomy techniques under controlled cadaveric conditions.

### 2.3 Tracheotomy procedure

The olecranon-type tracheotomy and expansion forceps were positioned parallel to the trachea’s long axis. The curved jaw tip was aligned perpendicularly to the skin over the trachea and inserted downward. The puncture depth was determined based on the distance measured by ultrasound, with an additional 1 cm added to ensure penetration of the trachea. Upon reaching the predetermined depth, the forceps were gradually rotated perpendicular to the skin so that the jaw tips shifted from vertical to horizontal alignment, minimizing the risk of posterior tracheal wall injury. The jaws were then opened to a length of approximately 2 cm, allowing the sharp inner edge of the arc-shaped jaws to incise the tracheal ring and pretracheal soft tissue longitudinally. An endotracheal tube was inserted between the open jaws and gradually advanced into the trachea. The forceps were then carefully withdrawn, and the inner core of the endotracheal tube was removed. The cuff was inflated, the tube was secured, and it was connected to a ventilator. The time required for the entire procedure was recorded.

After surgery, the endotracheal tube was removed, and a bronchoscope was used to examine postoperative airway damage and measure the incision length.

**FIGURE 3:**
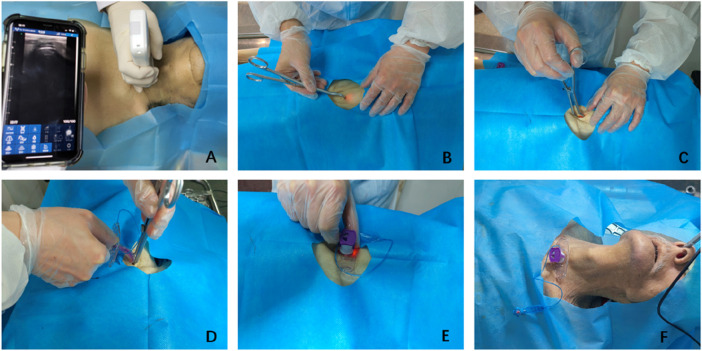
Surgical procedure. **(A)** Ultrasound examination to determine the puncture point. **(B)** Puncture into the trachea. **(C)** Reaching the predetermined depth and opening the forceps. **(D)** Inserting the tracheostomy tube into the trachea between the jaws. **(E)** Withdrawing the forceps. **(F)** Securing the tracheostomy tube.

Fiberoptic bronchoscopy was performed to assess damage to the tracheal wall following the procedure (Figure 3).

### 2.4 Outcome measures

General measures: age at death, sex, Body mass index (BMI), and tracheal depth (the vertical distance from the anterior wall of the trachea to the skin surface).

Surgery-related measures: operative time, incision length, and the time required for tracheal intubation following the incision.

### 2.5 Statistical analysis

Statistical analyses were performed using SPSS software (IBM Corp., Chicago, IL, United States). P < 0.05 was considered statistically significant. The normality of continuous variables was evaluated using the Shapiro-Wilk test. Data with a normal distribution were expressed as mean ± standard deviation (SD) and compared using the independent-sample t-test, whereas non-normally distributed data were expressed as median (interquartile range) and analyzed using the Mann-Whitney U test. Categorical variables were presented as frequencies and percentages, and intergroup comparisons were performed using the Chi-squared test.

## 3 Results

**FIGURE 4:**
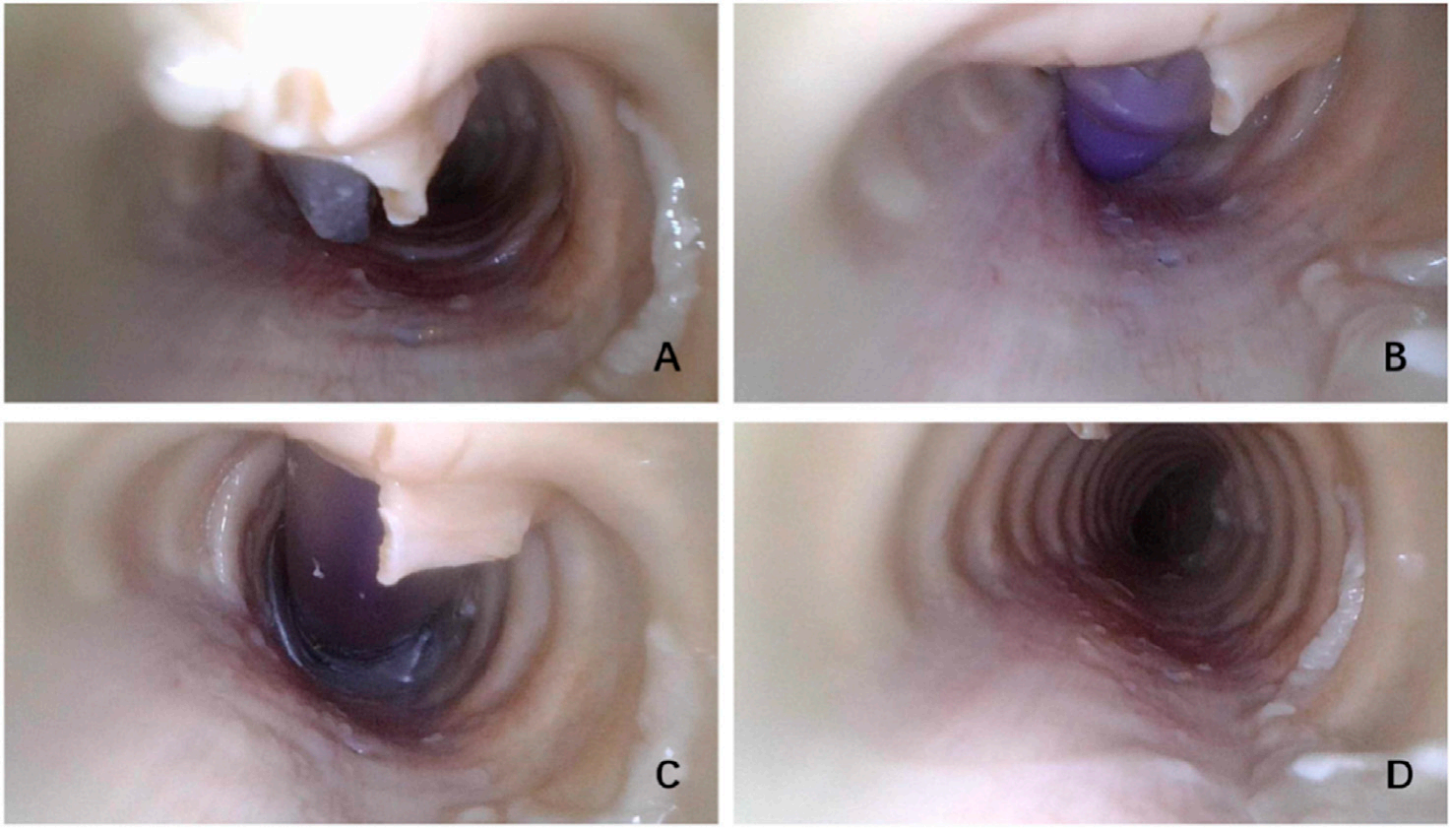
**(A)** Puncture into the trachea. **(B)** Tracheotomy tube insertion into the trachea. **(C)** Completed tracheotomy. **(D)** Postoperative examination of tracheal wall damage.

Tracheotomy was successfully completed on all 24 cadavers. Fiberoptic bronchoscopy confirmed no damage to the tracheal wall (Figure 4).

No significant differences were observed between the two groups in terms of age at death, sex, BMI, tracheal depth, or the time required for tracheal intubation following the incision (P > 0.05) (Table 1).

**TABLE 1:** Comparison between OTEF group and PDT group.

Group	OTEF	PDT	t/χ^2^	P
Age at death(years)	68.83 ± 5.98	69.17 ± 6.57	−0.133	0.896
Sex(Male/Female)	7/5	4/8	0.671	0.413
BMI(kg/m^2^)	19.52 ± 2.46	18.95 ± 2.78	0.53	0.62
Tracheal depth(mm)	11.48 ± 0.57	11.45 ± 0.73	0.112	0.91

**FIGURE 5:**
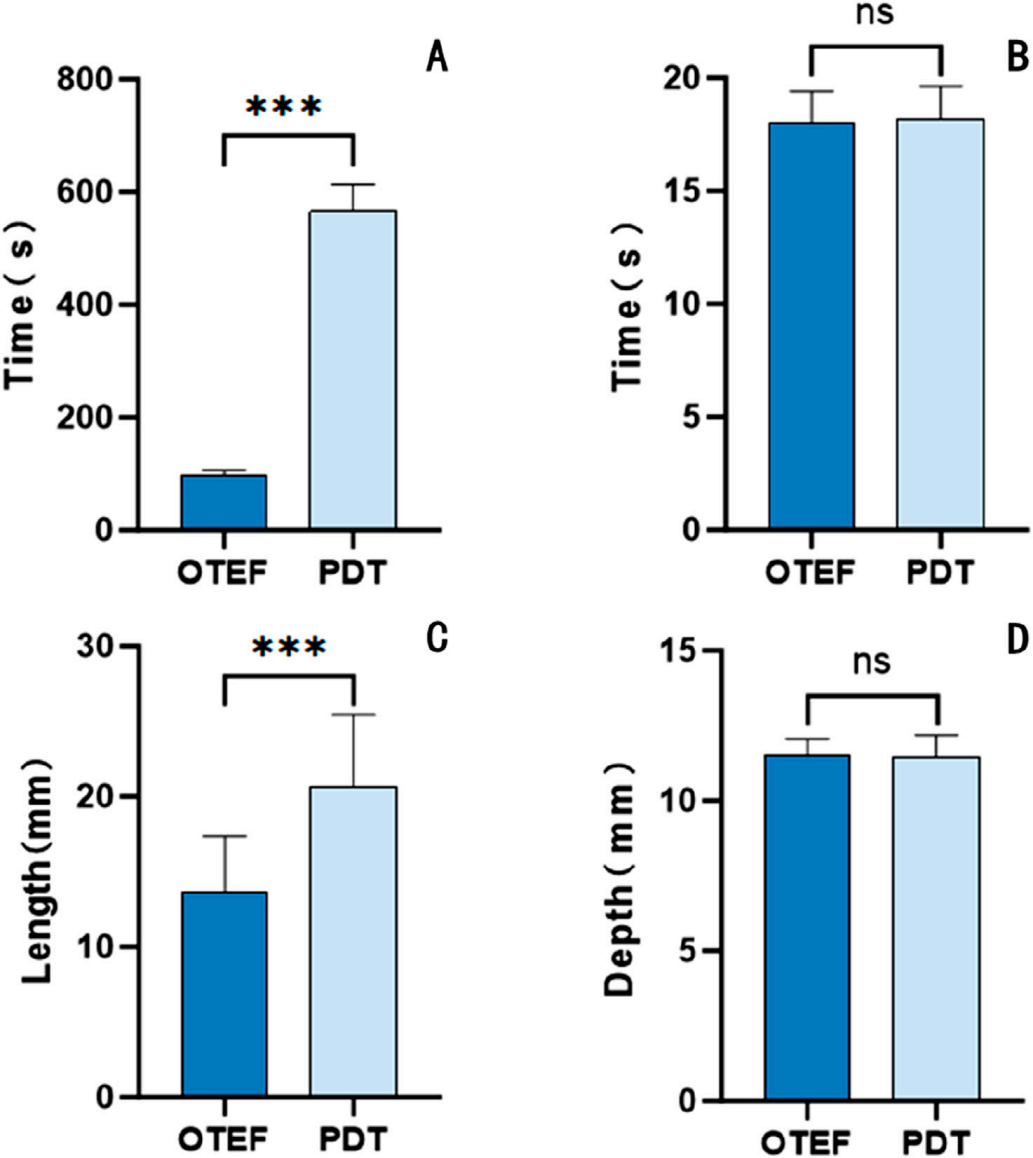
**(A)** Operation time: The mean operation time was 96.83 ± 8.82 s in the OTEF group and 566.50 ± 47.14 s in the PDT group, with a statistically significant difference (P < 0.001). **(B)** Time to tracheotomy tube insertion: The mean time was 18.00 ± 1.41 s in the OTEF group and 18.17 ± 1.47 s in the PDT group, with no significant difference (P > 0.05). **(C)** Incision length: The mean incision length was 13.67 ± 3.67 mm in the OTEF group and 20.67 ± 4.76 mm in the PDT group, showing a significant difference (P < 0.001). **(D)** Tracheal depth: The mean depth was 11.48 ± 0.57 mm in the OTEF group and 11.45 ± 0.73 mm in the PDT group, with no significant difference (P > 0.05).

In the OTEF group, the mean tracheotomy completion time was 96.83 ± 8.82 s, with a mean incision length of 13.67 ± 3.67 mm. In the PDT group, the mean tracheotomy completion time was 566.50 ± 47.14 s, and the mean incision length was 20.67 ± 4.76 mm. Compared with the PDT group, the OTEF group demonstrated significantly shorter operative times (P < 0.05) and smaller incision lengths (P < 0.05) (Figure 5).

## 4 Discussion

In resource-limited, understaffed, or emergency situations, a single-person tracheotomy represents a crucial option. Emergency tracheotomy performed by a single operator is a straightforward, rapid, and safe procedure to establish an airway in minimal time, potentially saving the patient’s life. Cadaveric experiments investigating single-person emergency tracheostomies provide essential insights into the feasibility, operational challenges, and limitations of this technique.

Olecranon-type tracheotomy and expansion forceps feature sharp anterior ends that efficiently penetrate the trachea with minimal tissue damage. Once the forceps’ tips are inserted vertically into the trachea, tilting the body of the forceps toward the patient’s head naturally directs the tips toward the feet. The device’s scale allows precise puncture depth determination, minimizing the risk of airway injury. The curved inner surface of the forceps includes a blade to cut through skin, muscle, and tracheal rings, while the outer blunt surface prevents damage to adjacent tissues during the procedure, emulating the incision process of a traditional tracheotomy scalpel. After the tracheotomy is completed, the jaw of the forceps guides accurate insertion of the tracheal tube into the trachea, facilitating the operation for healthcare providers.

The forceps tip is divided into two parts, front and back, which do not overlap like scissors. Only the front edge of the front part is sharp. The rear edge of the front part and the front edge of the back part form a smooth curved surface. The tracheotomy tube is inserted into the trachea between these two parts. Care must be taken during the operation to ensure the correct insertion position to minimize the risk of damaging the air cuff.

Traditional emergency tracheotomy methods, such as open surgical tracheotomy and cricothyrotomy, often require multiple operators, advanced training, and numerous instruments. These methods are time-consuming and associated with complications like incorrect incision placement, bleeding, and infection, particularly in suboptimal conditions. Cricothyrotomy is comparatively simpler for non-medical personnel to perform, as it involves fewer vital anatomical structures. Consequently, many military first-aid protocols prioritize cricothyrotomy as an emergency airway intervention on the battlefield. However, cricothyrotomy cannot provide a stable, long-term airway in patients with severe airway injuries, necessitating tracheotomy within 48 h to minimize postoperative complications and enhance recovery (Isabel Araújo et al., 2014; Trouillet et al., 2011; Yue et al., 2012; Rumbak et al., 2004). In contrast, the novel olecranon-type device simplifies the tracheotomy procedure through guided steps, reducing the technical and cognitive burden on operators. Cadaveric studies have shown that this device ensures consistent tracheotomy tube placement, minimizes variability, and enhances procedural safety. In this study, the incision length was 13.67 ± 3.67 mm, and the procedure was completed in 96.83 ± 8.82 s. According to literature, trained operators can perform emergency cricothyroidotomy on cadavers within 50 s (Schaumann et al., 2005). The findings suggest that olecranon-type tracheotomy and expansion forceps could replace emergency cricothyroidotomy in battlefield first aid. Following successful puncture, initial relief from suffocation was achieved.

Suffocation is common during emergencies, often due to trauma. Determining the puncture site can be challenging, particularly in cases involving unclear anatomical structures, such as in morbidly obese patients. Guinot et al. (2012). demonstrated that ultrasound-guided percutaneous dilation tracheotomy effectively resolves these issues by visualizing the trachea and tracheal rings, assisting operators in determining the puncture site and depth, and avoiding injury to blood vessels or the thyroid isthmus (Alan et al., 2015). The usefulness of ultrasonography in percutaneous dilational tracheotomy has been demonstrated in four open randomized studies. Among 275 patients who underwent preoperative ultrasonography, 40 (14.5%) experienced complications. In comparison, 285 patients who did not undergo ultrasonography had a higher complication rate, with 74 (26%) experiencing at least one complication. The use of ultrasonography significantly improved the success rate of first-attempt surgeries (Rudas et al., 2014; Yavuz et al., 2014; Ravi and Vijay, 2015; Gobatto et al., 2016). Additionally, some studies have reported that fiberoptic bronchoscopy can also reduce the occurrence of complications during tracheotomy (Saritas et al., 2016). However, fiberoptic bronchoscopy is challenging to operate, requires significant training, and is prone to damage, limiting its practicality in complex first-line emergency environments. In this study, portable ultrasound exploration was used to assist operators in accurately identifying the trachea before tracheotomy. The time from exploration initiation to operation completion was 96.83 ± 8.82 s. Portable ultrasound devices enhance operator accuracy and reduce complications such as pneumothorax, airway injury, and detubation. Furthermore, their portability and ease of use make them suitable for prehospital settings, remote locations, and emergency situations.

The integration of tracheal ultrasound image recognition and artificial intelligence enables the real-time annotation of anatomical structures like the trachea and thyroid in ultrasound images. This, to a certain degree, alleviates the difficulty of related training. Nevertheless, it is of utmost importance to closely monitor the systemic risks associated with artificial intelligence and guard against the emergence of blind trust.

The olecranon-type device is particularly useful in prehospital care, remote areas, and disaster scenarios where medical teams may be unavailable. It has potential applications in military medical kits and first-aid equipment for extreme environments, mass casualty events, and pandemics. By enabling single operators to perform tracheotomies efficiently, this device may improve survival rates and reduce complications associated with delayed airway management. The integration of portable ultrasound machines with this device further supports accurate tracheal localization and enhances procedural safety.

This study demonstrated the feasibility and safety of the olecranon-type tracheotomy and expansion forceps (OTEF) for single-operator tracheotomy under cadaveric conditions. However, several limitations should be acknowledged. First, the present study was conducted on cadaveric models, which cannot fully replicate the physiological conditions of living subjects, such as tissue elasticity, bleeding, and airway movement. Consequently, the potential risks of vascular or thyroid injury, as well as posterior tracheal wall damage, remain to be verified in future animal and clinical studies.

In addition, although no statistically significant difference in sex distribution was observed between the two groups (p = 0.413), potential anatomical variations related to sex—such as differences in neck circumference, tracheal diameter, and soft tissue thickness—may influence procedural performance. These factors could affect the ease of tracheal identification and puncture accuracy during the tracheotomy procedure. Future studies with larger and more balanced samples are warranted to further investigate the impact of sex-related anatomical differences on procedural outcomes.

Furthermore, operator-related factors such as learning curve, clinical background, and training duration were not analyzed in the present study. We plan to explore these parameters in future work to better define the learning requirements and skill acquisition process for single-operator tracheotomy. The learning curve for using this device must also be considered. While designed for simplicity, effective use requires proper training to ensure operators can confidently and safely perform the procedure under stress. Standardized training protocols, including simulation-based modules, are critical to the device’s widespread adoption.

In future studies, we plan to conduct a systematic investigation of the learning curve associated with single-operator tracheotomy. Although the present study primarily focused on the feasibility and procedural safety of the olecranon-type tracheotomy and expansion forceps (OTEF), the influence of operator experience and training duration on procedural performance remains to be fully elucidated. Understanding the learning curve is essential for defining the minimum training requirements, optimizing teaching strategies, and improving overall procedural safety and efficiency. To achieve this, future work will include quantitative assessment of performance indicators-such as operation time, success rate, and procedural accuracy-cross operators with varying levels of clinical experience. These data will help establish a standardized training protocol for the single-operator tracheotomy technique and provide valuable evidence for its wider clinical implementation.

## Data Availability

The original contributions presented in the study are included in the article/supplementary material, further inquiries can be directed to the corresponding authors.
